# Supplementary material for: DMRforPairs: identifying Differentially Methylated Regions between unique samples using array based methylation profiles
Source: BMC Bioinformatics. 2014 May 15;15:141. doi: 10.1186/1471-2105-15-141 (PMC4046028; doi:10.1186/1471-2105-15-141)
Supplement: Additional file 2 — DMRforPairs output for the comparison of A431-MCF7 and NA17018-NA17105. Please start from the HTML files in each folder. Available via the BMC Bioinformatics website. [file 1471-2105-15-141-S2.zip › 1394847754114233_MOESM2_ESM/A431_MCF7/figures/6867.pdf]

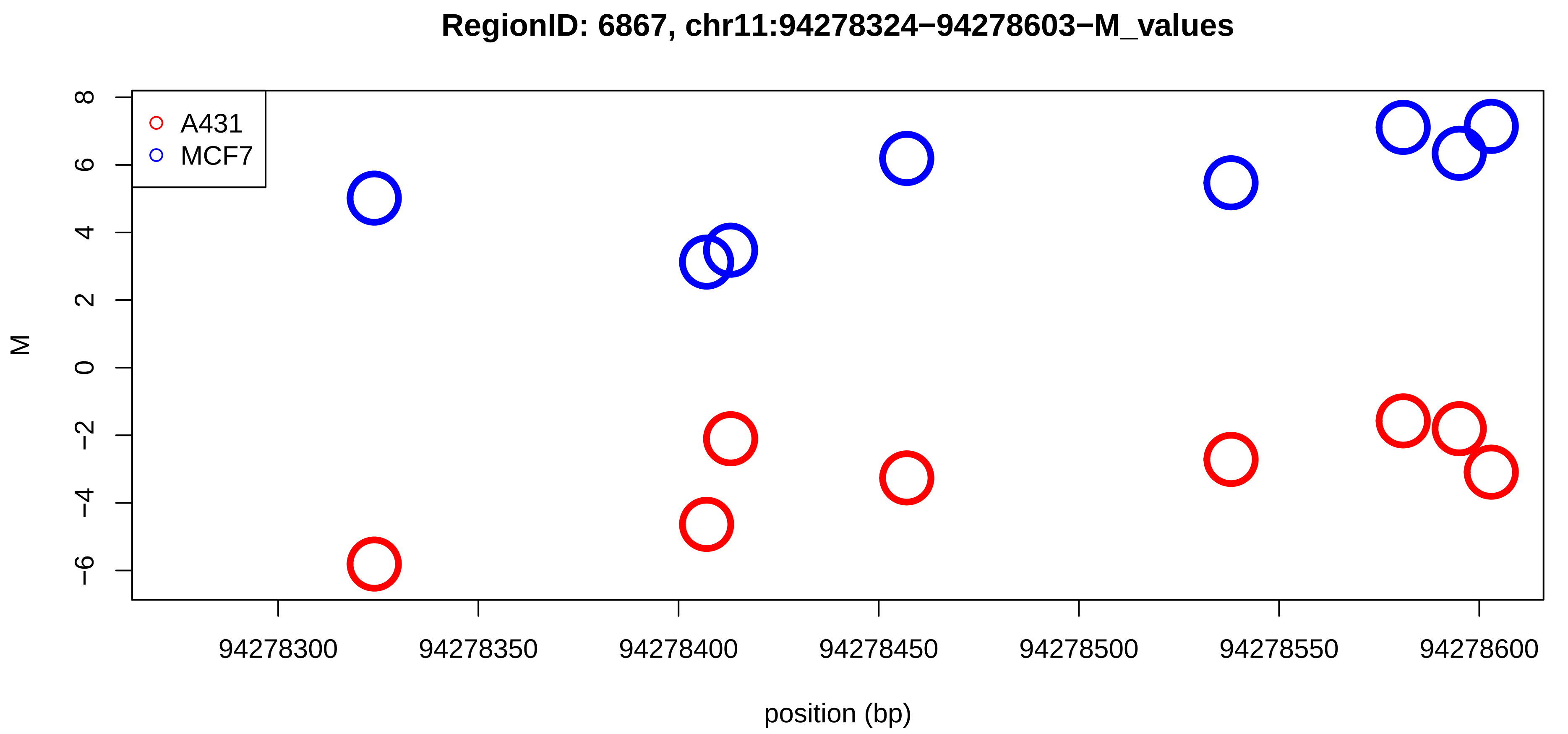
RegionID: 6867, chr11:94278324−94278603−M_values
A431
MCF7
M
position (bp)
94278300
94278350
94278400
94278450
94278500
94278550
94278600

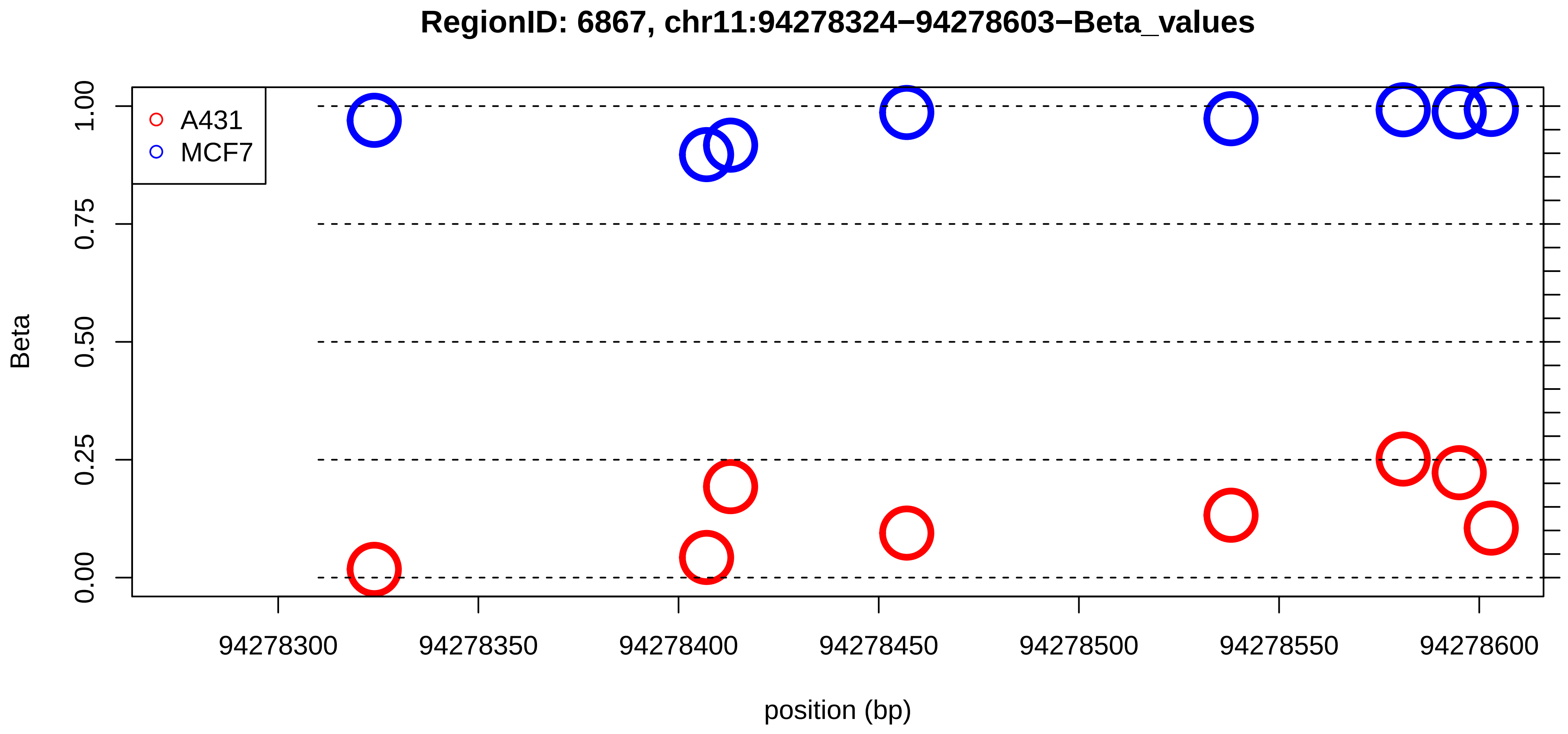
RegionID: 6867, chr11:94278324−94278603−Beta_values
A431
MCF7
Beta
position (bp)
94278300
94278350
94278400
94278450
94278500
94278550
94278600

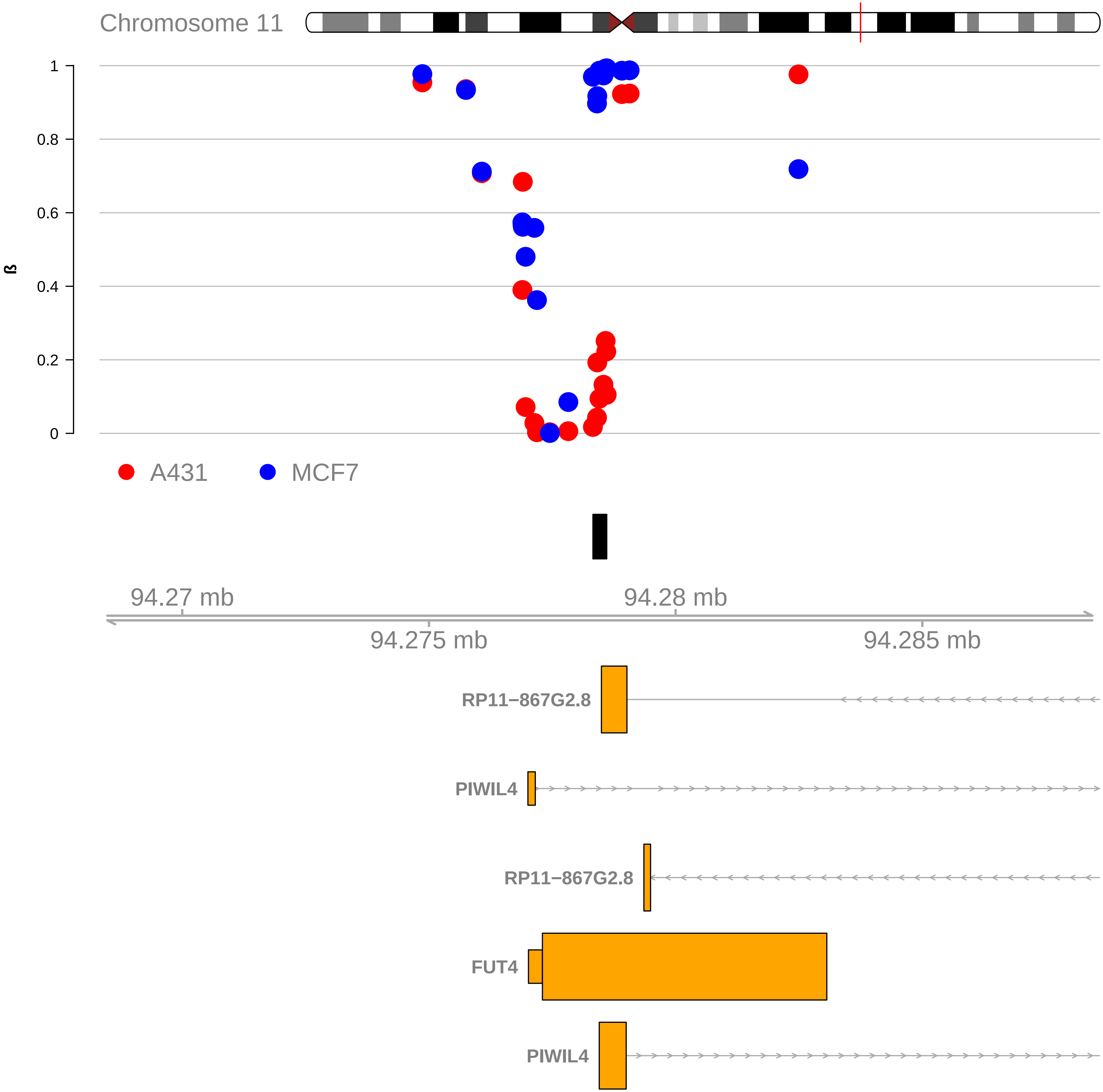

Chromosome 11
ß
1
0.8
0.6
0.4
0.2
0
A431
MCF7
94.27 mb
94.28 mb
94.275 mb
94.285 mb
RP11−867G2.8
PIWIL4
RP11−867G2.8
FUT4
PIWIL4
